# A proposed analytical approach to estimate excess daily mortality rates in Ecuador

**DOI:** 10.3389/fpubh.2024.1250343

**Published:** 2024-03-08

**Authors:** Raul Patricio Fernandez-Naranjo, Jorge Vasconez-Gonzalez, Juan S. Izquierdo-Condoy, Samanta Landazuri, Diana Castillo, Esteban Ortiz-Prado

**Affiliations:** One Health Research Group, Faculty of Medicine, Universidad de las Americas, Quito, Ecuador

**Keywords:** excess deaths, COVID-19, Ecuador, statistical bootstrapping, death toll

## Abstract

**Background:**

The COVID-19 pandemic has proved deadly all over the globe; however, one of the most lethal outbreaks occurred in Ecuador.

**Aims:**

This study aims to highlight the pandemic’s impact on the most affected countries worldwide in terms of excess deaths *per capita* and per day.

**Methods:**

An ecological study of all-cause mortality recorded in Ecuador was performed. To calculate the excess deaths relative to the historical average for the same dates in 2017, 2018, and 2019, we developed a bootstrap method based on the central tendency measure of mean. A Poisson fitting analysis was used to identify trends on officially recorded all-cause deaths and COVID-19 deaths. A bootstrapping technique was used to emulate the sampling distribution of our expected deaths estimator ${\overset{⌢}{\mu }}_{deaths}$ by simulating the data generation and model fitting processes daily since the first confirmed case.

**Results:**

In Ecuador, during 2020, 115,070 deaths were reported and 42,453 were cataloged as excess mortality when compared to 2017–2019 period. Ecuador is the country with the highest recorded excess mortality in the world within the shortest timespan. In one single day, Ecuador recorded 1,120 deaths (6/100,000), which represents an additional 408% of the expected fatalities.

**Conclusion:**

Adjusting for population size and time, the hardest-hit country due to the COVID-19 pandemic was Ecuador. The mortality excess rate shows that the SARS-CoV-2 virus spread rapidly in Ecuador, especially in the coastal region. Our results and the proposed new methodology could help to address the real situation of the number of deaths during the initial phase of pandemics.

## 1 Introduction

The coronavirus disease 2019 (COVID-19) pandemic continues to put unprecedented pressure on countries and their health systema. As of December 2022, more than 644 million cases have been reported worldwide, and at least 6.6 million deaths have been officially registered as caused by COVID-19 (1, 2). The Severe Acute Respiratory Syndrome Coronavirus 2 (SARS-CoV-2) virus has mutated several times since the first genome was sequenced (1–4). Nowadays, the predominant circulating variants have increased their transmissibility and, even though their virulence seems to be less than previous variants, the constant influx of new cases results in a continuous state of alert, surveillance, and death, especially for the most vulnerable patients (5–8). In terms of morbidity, mortality, and health system impact, Latin America is the region most affected by the pandemic, with 16.4% of the total number of COVID-19 confirmed cases and 20.6% of the total number of confirmed COVID-19 deaths globally, while sharing only 5.5% of the global population (9, 10). Although these figures are alarming themselves, there is a hidden reality about the actual number of deaths from COVID-19 in several countries from Latin America, including Ecuador (11). As the region has limited diagnostic capabilities and struggles to manage the number of daily cases, the unprecedented pressure is overwhelming health systems. and COVID-19-related deaths in Peru, Honduras, Brazil, or Ecuador, where excess mortality is more representative than COVID-19 officially reported deaths, also tend to be underreported (12).

According to demographers, the best tool available during a pandemic or a massive natural disaster to estimate the number of deaths is excess mortality, defined as the difference between the observed number of deaths in specific periods and the expected number of deaths in the same period (13–15). Excess mortality may provide a more complete and timely indirect measure of mortality (16–18) by encompassing deaths from all causes; excess mortality exceeds the variation between countries in reporting and proof of COVID-19 and misclassification of cause of death-on-death certificates (19). The use of excess mortality is now widely used as a reporting tool. For instance, in England, a study by Sinnathamby et al. showed higher excess mortality from all causes during the current COVID-19 pandemic (20). An analysis on variations in the number of excessive mortalities among countries showed that, in the United States and Spain, around 25 and 35% excess mortality was not reflected in the official COVID-19 statistics, respectively (13). Some of the most significant evidence comes from South America, where countries such as Mexico, Peru, Brazil, and Ecuador have suffered a massive surge in cases that have left thousands of deaths behind, not all registered as COVID-19 (12, 21–23). In Ecuador, during the first 54 days, 474 COVID-19 confirmed deaths were officially reported; nevertheless, at least 4,780 deaths were reported as acute respiratory distress syndrome (ARDS) during the same period of time, suggesting an important underreporting and undertesting of COVID-19 cases in the country (23).

Several research groups have sought to determine the pandemic’s real impact using historical records and average deaths in previous years as a good approximation of the reality experienced by the pandemic, especially in developing countries. The approach has been based on cumulative deaths rather than daily *per capita* deaths (24–26). We propose an innovative approach that uses mean in the context of bootstrapped simulations to replicate the data generation mechanism of death time series and obtain more robust estimations of expected deaths to quantify excess mortality in Ecuador to recognize the real impact and death toll attributable to COVID-19.

The limitations of a classical method, where the mean is used to estimate excess mortality, are mainly related to possible bias due to the lack of data (a standard issue in most of the reports on COVID-19 cases and deaths around developing countries) and sub-estimation errors. In addition, missing values could mislead estimations by increasing the sample to compute the mean and then calculate the excess mortality.

On the other side, the proposed approach based on bootstrapping mitigates the previous issues by sampling for all available values and then computing the mean, which is called bootstrapped means. The goal of bootstrapped means when using this method is to replicate with more considerable accuracy the exact distribution of deaths so that the computed mean is closer to an accurate approximation of the value and then the excess of mortality is more credible rather than only using the classic mean over a series of given values.

The aim of this work is to highlight the pandemic’s impact on the most affected countries worldwide in terms of excess deaths *per capita* and per day.

## 2 Methods and data

### 2.1 Study design

An ecological study of all-cause mortality recorded in Ecuador during the most lethal COVID-19 wave in 2020 was performed. All deaths recorded within the national registry database in Ecuador were used for COVID-19 and non-COVID-19 related deaths during the first year.

### 2.2 Setting

The study was carried out in Ecuador, one of the smallest Latin-American countries located on the equatorial line and bordering the Pacific Ocean. Ecuador shares borders with Peru and Colombia, and its current population is estimated to be 17,577,116 inhabitants. The country has four regions (Coast, The Highlands, The Amazonian, and the Galapagos Islands) 24 provinces, and 221 political subdivisions called cantons (cities).

### 2.3 Population

Our study included all nationwide recorded deaths from 2017 to 2020. A total number of 115,070 deaths in 2020 were analyzed; 42,453 of those were cataloged as excessive deaths.

### 2.4 Variables

The data retrieved regarding deaths in Ecuador had the following variables: jurisdiction (canton, province, and region), date, and total absolute and relative number of deaths from 2017 to 2020. Total deaths represent the number of deaths in each specific period considered in the analysis. For other complementary analyses, variables such as region or contagious cases were used, which were obtained from the same official websites.

### 2.5 Data source / measurement

Data for this study was obtained using the free information available from historical databases of the National Institute of Statistics and Census (INEC) located within the following freely available repository (27);^1^ all cases registered in this base were confirmed cases of COVID-19 (ICD-10:U07), with data from January 2017 to December 2020. Mean was computed at different periods, and then the difference of values between deaths during the COVID-19 pandemic was compared against average deaths during 2017–2019. The same method was applied for the bootstrapped concept. However, in this case, we simulate deaths’ behavior considering what would happen in other years if having an extreme event such as a pandemic to model the generation mechanism of data for 2020.

### 2.6 Bias

To reduce the risk of bias or unvoluntary errors, two researchers retrieved the data separately. Once data was downloaded, both investigators analyzed the dataset separately. The researchers resolved any questions or doubts after reaching consensus with a third researcher included in the analysis. Means and confidence intervals were computed independently, instead of using the same R data code used for the entire analysis to confirm the homogeneity of the dataset used by both researchers. The only kind of bias we could find in this analysis is related to the quality of data. There were days where values changed due to administrative mistakes. In this way, if we had used only the mean to compute the excess of mortality, the computed values would be extremes. On the other hand, using the bootstrapped mean to compute excess alleviates this issue and makes estimations more accurate.

### 2.7 Study size

Excess deaths were calculated with a daily, weekly, and monthly resolution. Data of death cases at the monthly level was composed of a time series of 1,152 observations across 24 provinces of the country. At the weekly level, the time series of deaths was composed of 5,088 observations for 24 provinces. At the daily level, the time series of deaths was composed of 35,064 observations for 24 provinces. The time series starts on January 1, 2017, and ends on December 31, 2020. For cantons, the time series of deaths had 323,611 observations.

### 2.8 Statistical methods

Descriptive statistics were applied to describe differences among provinces and cantons. To analyze the evolution of deaths, we initially applied dynamic statistical tests to the daily death series in each province as well as across the whole of Ecuador to identify on which days there were changes in the behavior of the number of reported cases. Excess deaths were computed for all days available, since the goal of this work is to show how the computed excess can differ in the way the methods are used.

In all provinces, we have n daily observations. Each i observation from two to n was used as a change point. With this reference point, the previous and subsequent observations constitute different datasets. Then, a variance test was applied to identify the variability and test the following hypothesis:

$$H_{0}:Deathsbefore\;i\;are\;equaltoDeathsafter\;i$$

$$H_{1}:Deathsbefore\;i\;are\;differenttoDeathsafter\;i$$

As no data was available before the first day in the death series, we started from i=2 . We obtained a series of *p*-values for each i and therefore selected the minimum of those where $H_{0}$ is rejected. This point highlights where an important change occurred.

The Poisson adjustment makes it possible to identify what the trend in the evolution of the cases of death will be like (28). Based on a Poisson distribution, it measures the increase or decrease considering the change rate in death cases by days.

Given the data series of deaths, we computed mean in the period time mentioned. Then the value reported each day, week, or month is contrasted against this estimate to compute the excess.

After, we applied sampling with replacement over it for n number of times. From each time we computed the mean. We stored the mean in an array. Then we computed the mean of all saved estimates as well as quantiles to create intervals. We took the bootstrapped mean from n simulations and contrasted reported deaths again for each day, week, or month. With these results, we computed excessive mortality.

To calculate the excess deaths at the country and province level, we developed a bootstrap method based on the central tendency measure, mean, which is used for many studies and clinical investigation centers to calculate excess mortality.

Statically, the mean is used to make exploratory analysis, and the measure is sensitive to extreme values (29). Consequently, this might impact the quality of results. Moreover, due to the current pandemic, all countries are experiencing many deaths per day and comparing them with the traditional values of the deaths series of previous years could inflate the excess deaths indicator.

To avoid extreme estimates in the expected deaths, we used a bootstrapping approach. The essential concept of bootstrapping is to emulate the repetition of certain experiments by simulating new data, followed by a statistical measure’s recalculation using such simulated data (30).

The bootstrap emulates the sampling distribution of our expected deaths estimator ${\overset{⌢}{\mu }}_{deaths}$ by simulating the data generation and model fitting processes. It does this by generating artificial data $y^{\left(b\right)}=\left(y_{1}^{\left(b\right)},\ldots ,y_{n}^{\left(b\right)}\right)$ from a distribution that approximates the true unknown sampling distribution of the actual data. This is repeated several times, B , resulting in an extensive collection of bootstrap estimators $\mu_{deaths}^{\left(b\right)}$ , b=1,…,B . The distribution of these artificially generated bootstrap estimators can be used to infer the sampling distribution of ${\overset{⌢}{\mu }}_{deaths}$ .

As the true sampling distribution of the death time series is unknown, we will use nonparametric bootstrapping. Suppose the death data $y_{i}$ , i=1,…,n , are independent and have an identical distribution. In that case, the empirical cumulative distribution (ecdf) can be used as a discrete approximation of the true cumulative function.

$${\overset{⌢}{F}}_{ecdf}\left(y\right)=\frac{1}{n}\overset{n}{\underset{i=1}{\sum }}I\left(y_{i}\leq y\right)$$

The general algorithm we define is as follows:

1. Generate a bootstrap sample from death data $y^{\left(b\right)}=\left(y_{1}^{\left(b\right)},\ldots ,y_{n}^{\left(b\right)}\right)$ $\hat{F}$ .
2.
$\mu_{deaths}^{\left(b\right)}$ using $y^{\left(b\right)}=\left(y_{1}^{\left(b\right)},\ldots ,y_{n}^{\left(b\right)}\right)$ .

With the results of simulations, we obtained ${\overset{⌢}{\mu }}_{deaths}$ and defined bootstrap confidence intervals at α=0.05 using the percentile method. We completed 1,000 bootstraps to retrieve robust estimates (30). Because data are available at the daily level, we produced monthly, weekly, and daily time-scale estimates for the country and its provinces.

## 3 Results

In Ecuador, since the beginning of the pandemic, at least 42,453 people have died in excess when compared with the previous year’s averages. The previously mentioned value comes from the classic mean definition for calculating excess deaths.

### 3.1 Maximum number of deaths per day

The maximum number of deaths in one single day in Ecuador occurred on 04/04/2020, with at least 1,120 deaths, having an excess in mortality in at least 921 deaths. In the case of Provinces of the Ecuadorian region, for Guayas, the maximum occurred on the same day, with 848 total deaths and 795 excess. In contrast, for the second national wave, Pichincha suffered the worst, having a total of 97 deaths in excess on 17/20/2020. As of the last update of our analysis (December 31, 2020), there were 101,439 deaths in Ecuador, with 42,453 excesses.

**Figure 1:**
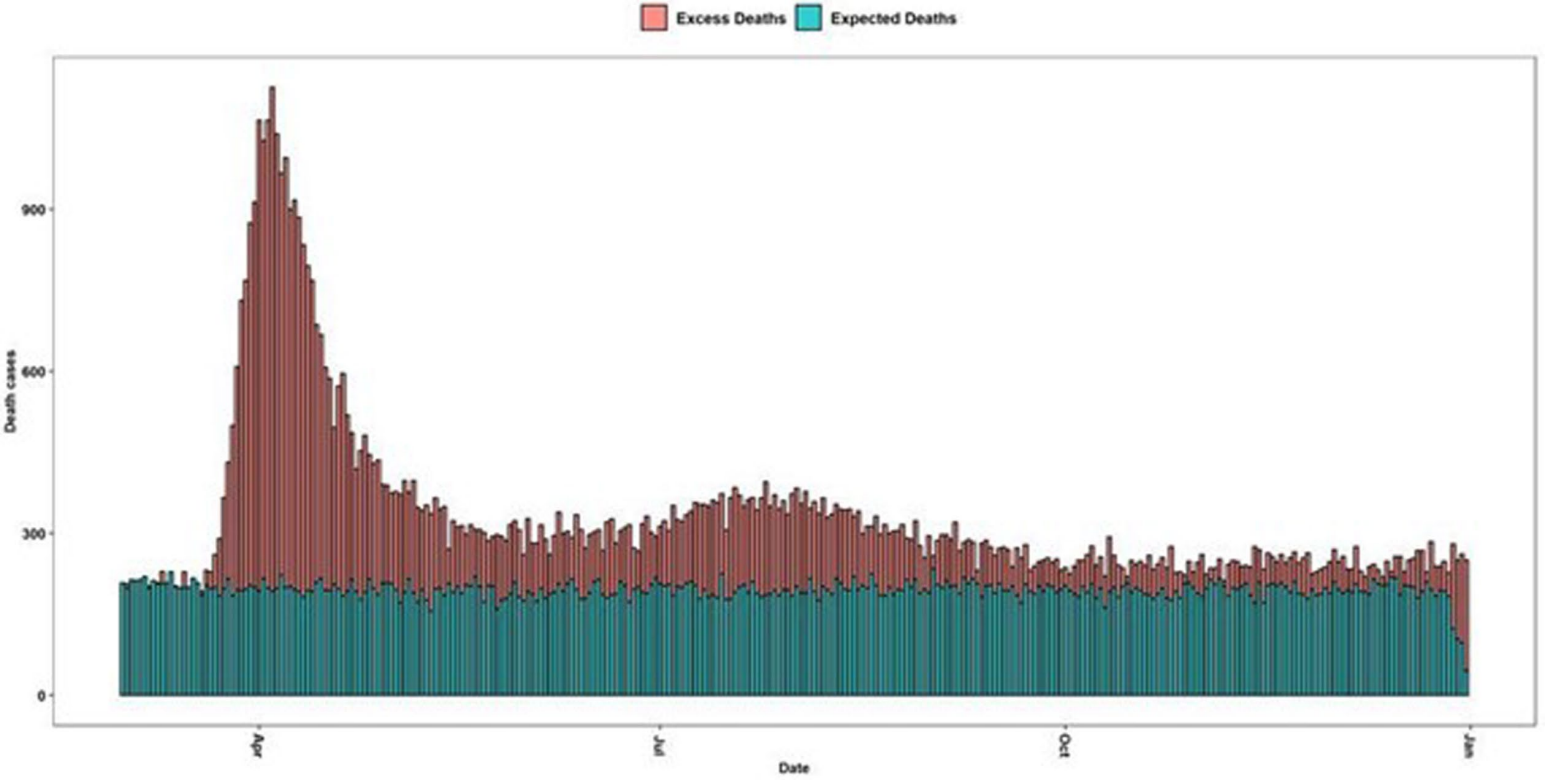
Maximum number of deaths per date in Ecuador. Green bars are the average deaths per day from 2017 to 2019 and the red-colored bars are the excessive mortality daily curve.

Our methodology based on bootstrap simulations derives an estimate of 30,213, which, compared with the classical method, calculated total excess deaths in 2020 at 42,453, which implies a difference of 12,240 death cases (Figure 1).

### 3.2 Excess mortality per province

**Figure 2:**
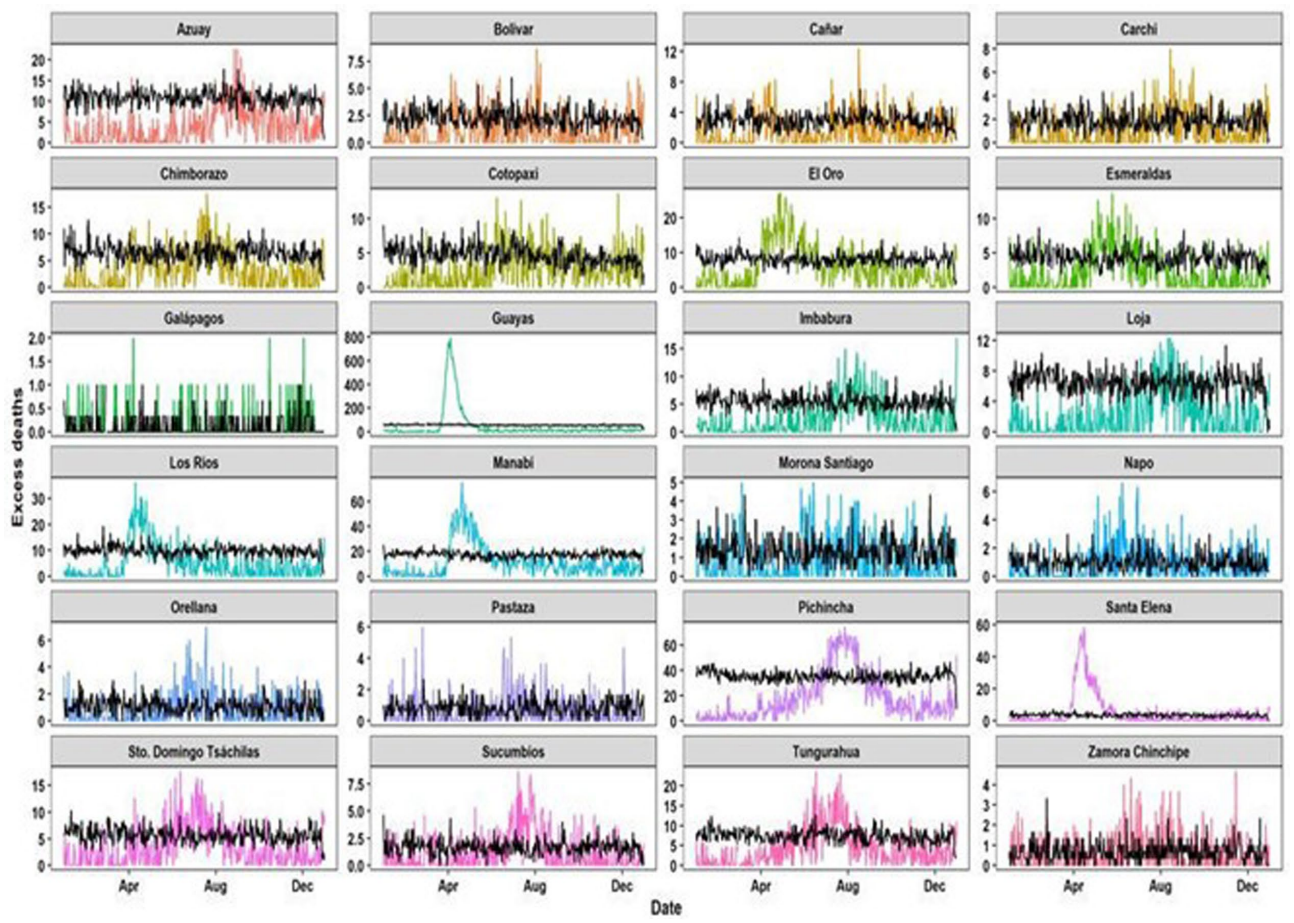
Daily excess deaths per Ecuadorian provinces during 2020 (Black lines are the normal behavior of deaths in previous years; Colored lines are the observed deaths in 2020).

In terms of provinces below our formulation, the cumulative excess on December 31, 2020, for Guayas was 10,727 deaths, with a maximum daily excess of 795, whereas for Pichincha it was 1,785 deaths, with a maximum daily excess of 72 deaths (Figure 2).

According to the provinces, the classical estimation for excess mortality was compared to the bootstrapped estimation up to December 31, 2020. The differences in values, compared with the classical excess approach, demonstrated that our method was more consistent than just using the mean as a measure to quantify excess deaths. For instance, Santa Elena had an increase of 87% regarding monthly excess deaths and an increase of 92% daily excess deaths (Table 1).

**Table 1:** Excess mortality per time comparing the classic estimation and the bootstrapped estimation (new).

Province	Monthly excess	Weekly excess	Daily excess	Monthly excess	Weekly excess	Daily excess	Monthly	Weekly	Daily
	Classic estimation			Proposed new estimation			Excess against deaths (%)		
Azuay	299	83	21	284	81	24	45%	50%	69%
Bolivar	52	20	9	46	20	8	38%	54%	80%
Cañar	72	31	12	79	31	12	45%	58%	80%
Carchi	69	29	8	74	27	7	54%	66%	78%
Chimborazo	245	69	15	230	66	16	51%	57%	70%
Cotopaxi	142	39	13	147	40	14	48%	53%	74%
El Oro	470	139	26	431	132	27	60%	67%	75%
Esmeraldas	198	50	12	176	49	12	55%	60%	71%
Galapagos	4	0	2	3	2	2	43%	67%	100%
Guayas	10,727	4,926	795	10,383	4,860	787	83%	91%	92%
Imbabura	209	66	13	209	63	14	54%	61%	70%
Loja	191	57	12	179	53	12	46%	53%	63%
Los Ríos	610	164	36	589	152	34	64%	67%	76%
Manabí	1,266	367	75	1,180	348	68	66%	72%	77%
Morona Santiago	41	12	5	34	13	5	44%	57%	83%
Napo	56	16	6	52	16	6	60%	67%	86%
Orellana	52	23	7	49	21	6	58%	72%	86%
Pastaza	38	12	6	38	12	6	58%	67%	86%
Pichincha	1,785	453	72	1,658	408	67	58%	60%	63%
Santa Elena	1,047	339	59	1,012	330	57	87%	91%	92%
Santo Domingo	266	77	18	238	67	18	55%	60%	75%
Sucumbíos	134	36	9	123	30	8	68%	70%	80%
Tungurahua	384	107	24	362	98	24	59%	63%	75%
Zamora Chinchipe	31	10	5	34	11	4	60%	69%	80%
Country Total	**15,009**	**5,826**	**931**	**14,057**	**5,764**	**889**	**67%**	**79%**	**79%**

### 3.3 Confirmed deaths against daily excess deaths by region

**Figure 3:**
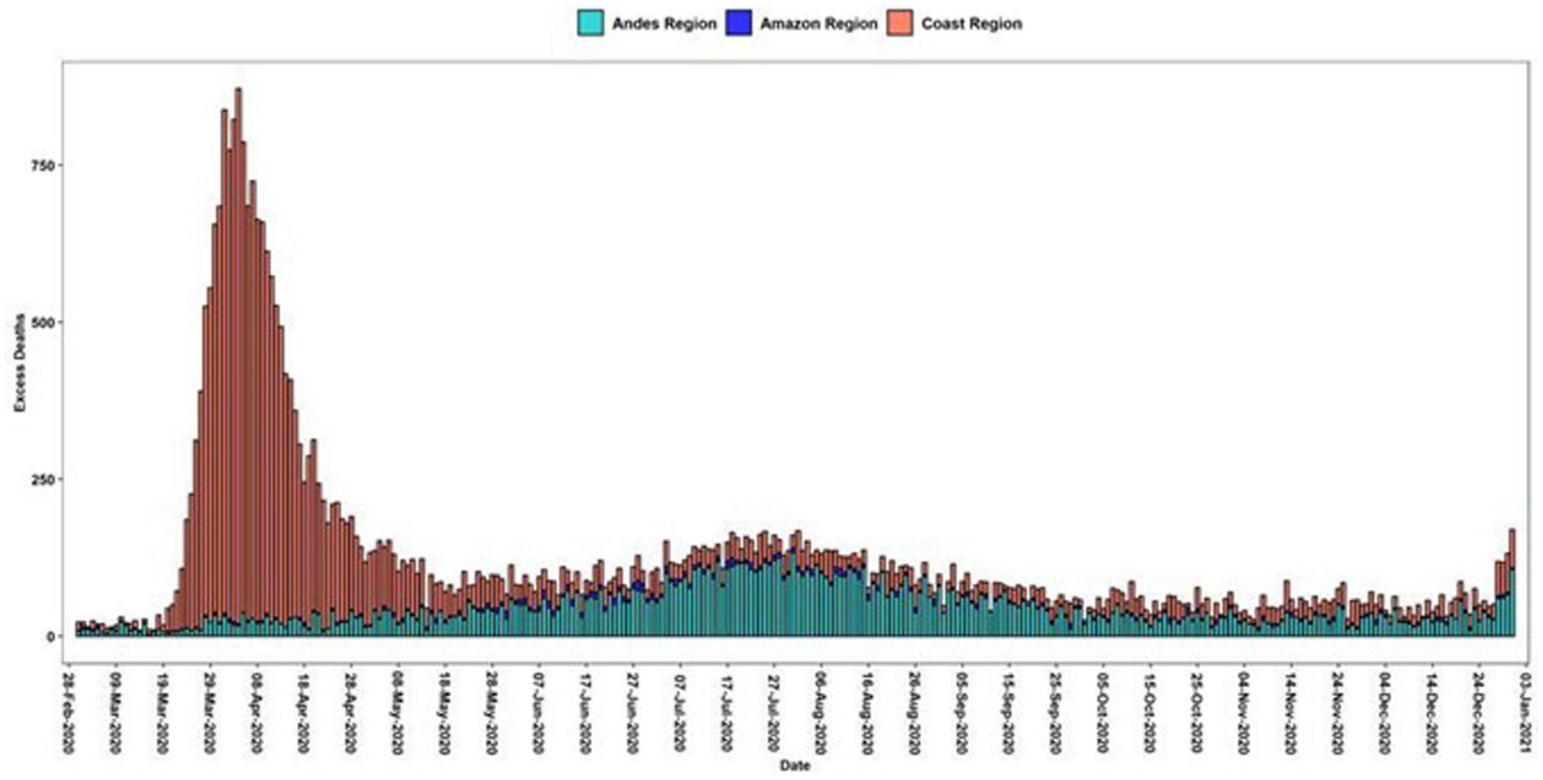
Daily excess deaths in Ecuadorian Regions. Green bars are the average deaths per day from 2017 to 2019 and the red-colored bars are the excessive mortality daily curve.

As an additional insight, using excess deaths below the classical estimation method as reported by the National Institute of Statistics and Census (INEC) in Ecuador, we compared the impact of daily excess deaths by regions, an analysis not previously performed in Ecuador (Figure 3).

Excess mortality is higher in the coast than in the highlands and the amazon region and the percentage increased during 2020 reached almost double that of the previous years (Table 2).

**Table 2:** Confirmed deaths against daily excess deaths by region in a year.

Region	Population	Total deaths	Expected deaths	Excess deaths	% increase	Rate per 100,000
Highlands	7,847,136	41,140	26,925	14,215	53%	181.1
Coast	8,631,859	57,293	30,042	27,251	91%	315.7
Amazon	956,699	2,965	1,982	983	50%	102.7
Galapagos	33,042	41	37	4	11%	12.1
Ecuador	**17,510,643**	**101,439**	**58,986**	**42,453**	**72%**	**242.4**

### 3.4 Confirmed deaths against daily excess deaths rates by province

Guayas was the province with the highest excess deaths, at 17,582, representing an increase of 103% over the expected deaths in comparison to previous years. This represented an excess mortality rate of 400.7 per 100,000 inhabitants. However, considering the variability of population density, the province of Santa Elena had the highest excess mortality rate, with 412.4 per 100,000 inhabitants (Table 3).

**Table 3:** Confirmed deaths against daily excess deaths by province.

Region	Province	Population	Total deaths	Expected deaths	Excess deaths	% increase	Rate per 100,000
Highlands	Azuay	881,394	4,425	3,266	1,159	35%	131.5
	Bolívar	209,933	951	673	278	41%	132.4
	Cañar	281,396	1,313	892	421	47%	149.7
	Carchi	186,869	850	576	274	48%	146.8
	Cotopaxi	488,716	2,240	1,403	837	60%	171.3
	Chimborazo	524,004	2,972	1,950	1,022	52%	195.0
	Imbabura	476,257	2,325	1,628	697	43%	146.2
	Loja	521,154	2,481	1,925	556	29%	106.6
	Pichincha	3,228,233	17,224	10,684	6,540	61%	202.6
	Tungurahua	590,600	3,618	2,216	1,402	63%	237.3
	Santo Domingo	458,580	2,741	1,712	1,029	60%	224.5
Coast	El Oro	715,751	4,422	2,439	1,983	81%	277.0
	Esmeraldas	643,654	1,871	1,279	592	46%	92.0
	Guayas	4,387,434	34,661	17,079	17,582	103%	400.7
	Los Ríos	921,763	4,465	2,962	1,503	51%	163.1
	Manabí	1,562,079	9,147	5,210	3,937	76%	252.0
	Santa Elena	401,178	2,727	1,073	1,654	154%	412.4
Amazon	Morona Santiago	196,535	541	396	145	37%	73.6
	Napo	133,705	477	306	171	56%	127.6
	Pastaza	114,202	340	253	87	34%	76.47
	Zamora Chinchipe	120,416	313	207	106	51%	88.3
	Sucumbíos	230,503	807	494	313	63%	135.6
	Orellana	161,338	487	326	161	50%	99.9
Galapagos Islands	Galápagos	33,042	41	37	4	11%	13.1
Ecuador		**17,510,643**	**101,439**	**58,986**	**42,453**	**72%**	**242.4**

**Figure 4:**
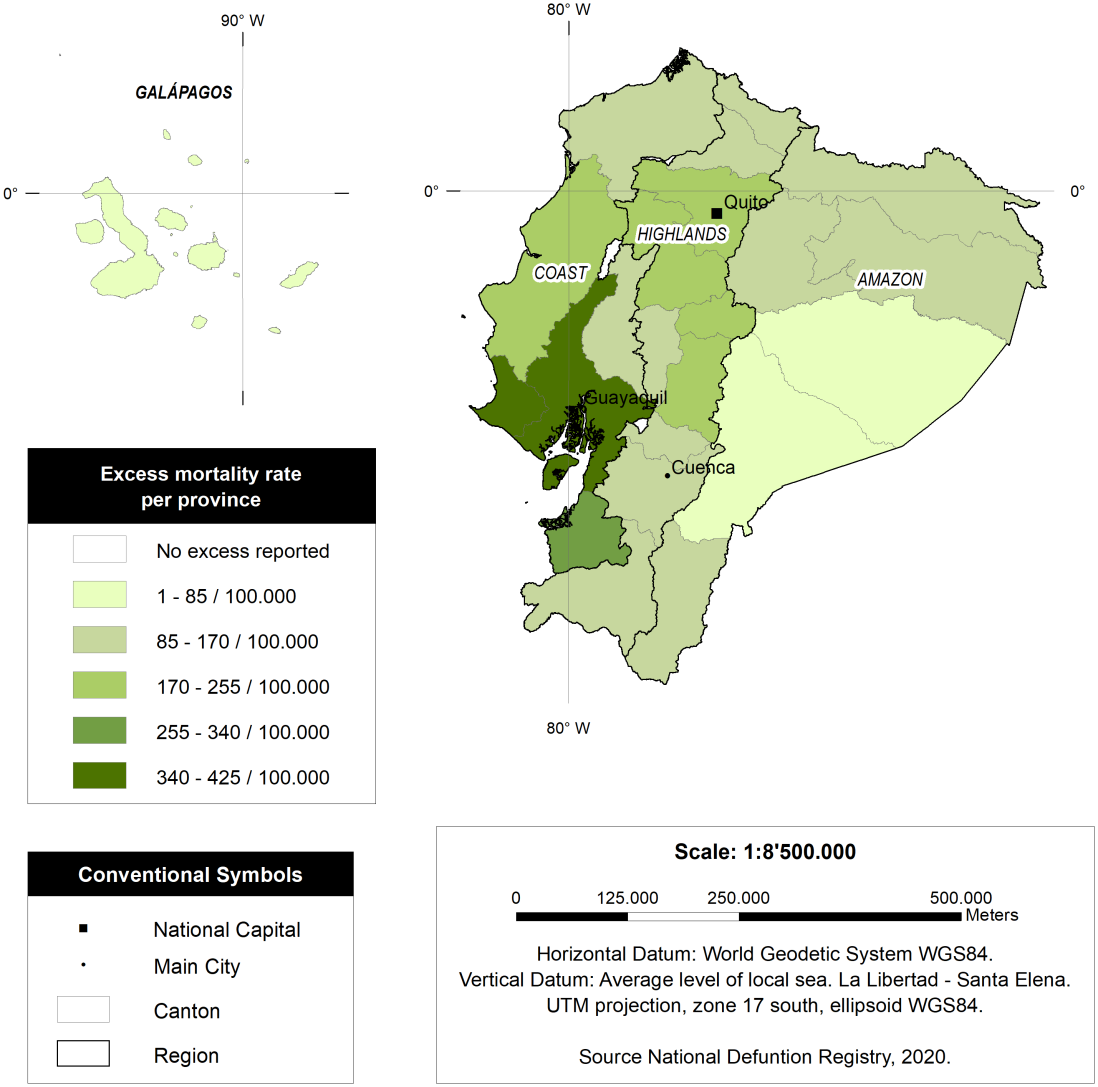
Excess mortality rate per province in Ecuador.

As observed in Figure 4, those provinces located in the coastal region of Ecuador have higher mortality *per capita* than those provinces and regions from the Amazon and the highlands.

### 3.5 Confirmed deaths against daily excess deaths rates by cantons

The cantons with the highest excess mortality rate were in the coastal region, with the provinces of Santa Elena and Guayas Provinces the most affected (Table 4).

**Table 4:** Confirmed deaths against daily excess deaths by cantons.

Rank	Canton	Total deaths	Expected deaths	Excess deaths	% Increase	Population	Rate x 100,000
1	Santa Elena	1,821	727	1,094	150%	188,821	579.2
2	Guayaquil	29,262	15,881	13,381	84%	2,723,665	491.3
3	Machala	2,812	1,516	1,296	85%	289,141	448.2
4	Manta	2,601	1,448	1,153	80%	264,281	436.3
5	Milagro	1,768	1,033	735	71%	199,835	368.0
6	Daule	1,145	516	629	122%	173,684	362.2
7	Santa Lucía	396	238	158	67%	45,004	351.8
8	Portoviejo	2,820	1,725	1,095	64%	321,800	340.4
9	Jipijapa	638	406	232	57%	74,645	310.8
10	Quevedo	1,591	940	651	69%	213,842	304.3
11	Ambato	3,200	2,032	1,168	57%	387,309	301.6
12	Durán	1,299	476	823	173%	315,724	260.6
13	Riobamba	2,147	1,492	655	44%	264,048	247.9
14	Latacunga	1,462	956	506	53%	205,624	246.2
15	La Libertad	635	348	287	83%	117,767	244.0
16	Pedro Carbo	411	288	123	43%	51,802	236.8
17	Santo Domingo	2,988	1,934	1,054	55%	458,580	229.9
18	Quito	18,017	11,880	6,137	52%	2,781,641	220.6
19	Azogues	696	508	188	37%	86,276	218.3
20	Ibarra	1,594	1,133	461	41%	221,149	208.3
21	Esmeraldas	1,397	949	448	47%	218,727	205.0
22	Pasaje	514	339	175	51%	87,723	199.1
23	Salinas	528	348	180	52%	94,590	189.9
24	Babahoyo	1,407	1,081	326	30%	175,281	186.2
25	Lago Agrio	650	428	222	52%	119,594	185.3
26	Rumiñahui	537	338	199	59%	115,433	172.7
27	Sucre	409	306	103	34%	62,443	165.0
28	Salitre	370	264	106	40%	65,765	160.7
29	Tulcán	591	431	160	37%	102,395	156.6
30	Samborondón	431	271	160	59%	102,404	156.2
31	Chone	767	566	201	36%	131,002	153.7
32	Montecristi	450	291	159	54%	107,785	147.2
33	Colta	312	247	65	26%	44,838	144.2
34	Cuenca	3,977	3,064	913	30%	636,996	143.3
35	Playas	373	288	85	29%	59,628	142.0
36	Guaranda	596	458	138	30%	108,763	126.6
37	Otavalo	582	441	141	32%	125,785	112.4
38	Tena	360	272	88	33%	79,182	111.6
39	Loja	1,699	1,403	296	21%	274,112	108.0
40	Empalme	395	303	92	30%	86,073	106.5

**Figure 5:**
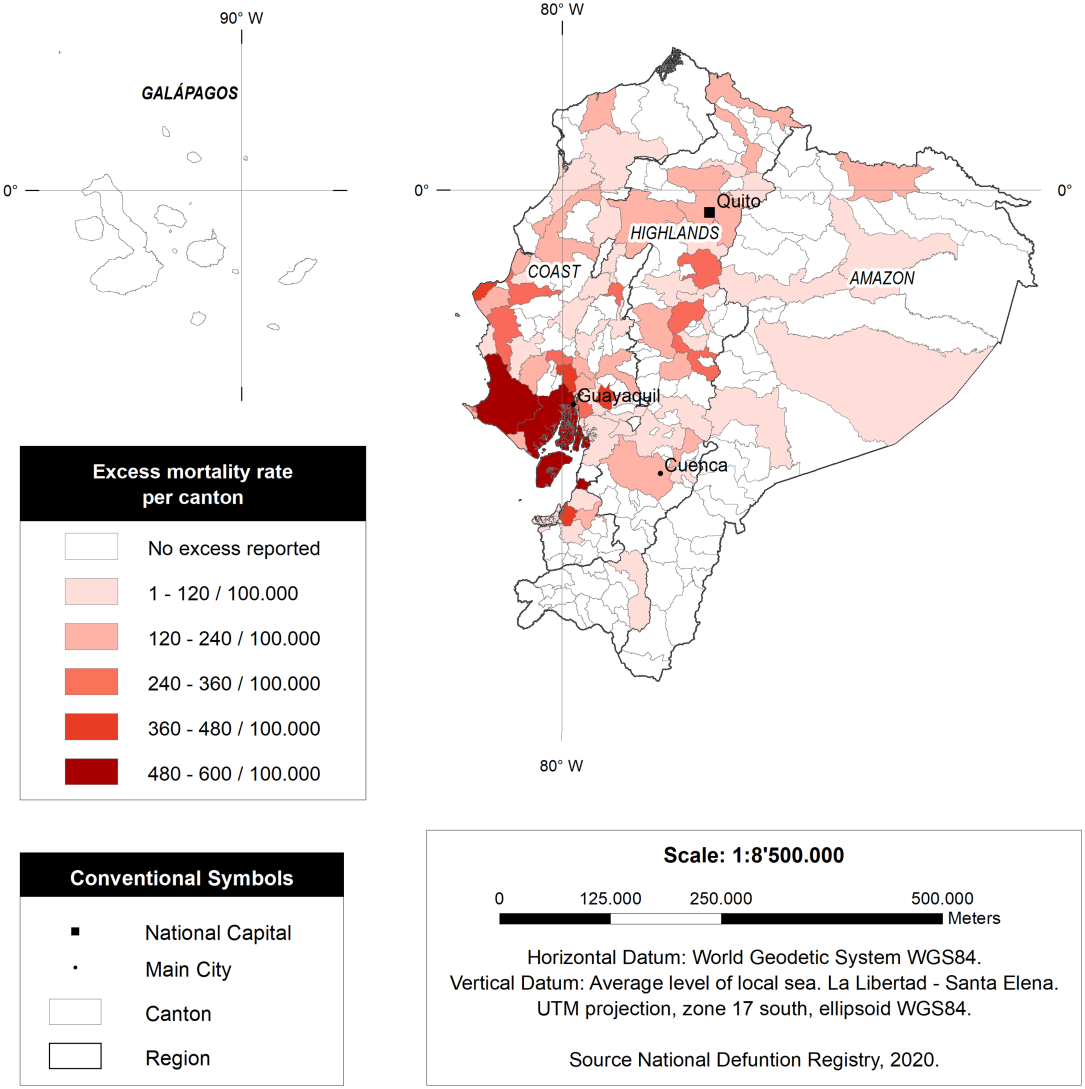
Excess deaths rate in Ecuador by canton in Ecuador.

Some cantons reached unprecedentedly high mortality rates. For instance, Santa Elena (a canton with the same name as the province) had 579.2 deaths per every 100,000 inhabitants, followed by Guayaquil with 491.3 per 100,000 inhabitants. At the same time, other cantons have significantly lower mortality rates, such as those located in the Amazon region or Galapagos (Figure 5).

## 4 Discussion

Ecuador has had the highest number of COVID-19 related excess deaths *per capita* reported in a single day worldwide. The impact of the pandemic during the early phase of the outbreak in the country was devastating (11, 23, 31).

While we know that some countries worldwide, such as the United States India, or Brazil, have reported higher daily COVID-19 related deaths, Ecuador exceeds those countries greatly when adjusting for its population (Table 5).

**Table 5:** Comparison of deaths attributed to COVID-19 in a single day in some countries of the region and the world (chart updated in March 2021).

Country	Total number of COVID-19 officially reported deaths	COVID-19 mortality crude /rate	Maximum number of deaths per day	% increases in terms of excess mortality	Highest mortality rate/100.000 per day
Ecuador	17,965	101	1,120	408%	6.27
Bolivia	12,731	108	84	256%	0.71
Peru	58,261	175	740	178%	2.21
Brazil	381,687	179	4,249	86%	1.98
Colombia	69,596	136	429	83%	0.83
Chile	25,532	133	316	68%	1.64
Mexico	214,957	168	1,584	60%	1.24
Argentina	60,083	132	515	40%	1.13
USA	586,152	178	5,057	47%	1.54
India	195,123	14	2,624	–	0.18

Such was the demand for hospital beds, medical attention, and medical supplies that, during the first wave of the pandemic in Ecuador, hundreds of critically ill patients were treated in their homes. This action resulted in painful scenes, with dozens of human bodies left on the streets while funeral homes were overwhelmed (23).

The government of Ecuador has only reported those deaths that were confirmed as COVID-19 cases. For instance, on August 29th, 2022, Ecuador reported 35,832 confirmed deaths due to COVID-19 and, for the same date, the excess mortality overpassed 89,418 deaths (27). Thus, 53,586 could represent the actual excess deaths in this period, at least 150% more than expected. This difference between excess deaths and the official COVID-19 deaths is similar to the data reported previously in Ecuador (11, 32, 33).

Our study capitalizes on the sole dataset available for Ecuador, offering a distinct analytical foundation. Employing historical mortality averages as a comparative baseline enhances methodological rigor, facilitating a precise and objective assessment of excess mortality attributable to the pandemic (34).

At the provincial level, it can be observed that significant outbreaks showed values higher or lower than those reported by the official method. Cevallos et al. estimated an interim excess death in Ecuador from March 17 to October 22, 2020; this indicated that excess deaths were estimated at 36,922 and also indicated that the peak in excess all-cause mortality in Ecuador may have occurred on April 4, 2020, with 909 excess deaths (35). However, this study did not analyze the excess of deaths at the provincial or canton level.

Comparative analysis with analogous Ecuadorian studies reveals a significant alignment in methodologies and outcomes. This consistency not only corroborates our approach but also augments the veracity of our findings. Methodological alignment with nationally recognized research underscores both the suitability of our techniques and the pertinence of our results within the Ecuadorian milieu, thereby reinforcing the scientific merit and contextual relevance of our study (24, 33, 34).

Our study also explores the epidemiological dynamics of COVID-19-related excess mortality with a cantonal resolution (Figure 5). For instance, Santa Elena was one of the most affected jurisdictions in the country, reaching an astonishing 579.2 excess deaths per 100,000, five times more than Italy or Spain during the worst part of their pandemic (25, 36); other countries with excess deaths lower than Ecuador were Germany and, in Latin America, Peru, Chile, and Boliva (37–40) It can be seen that most of the affected cantons during the pandemic belong to the Coastal region. This might be caused by its demographic density or triggered by cultural aspects linked to higher mobility; for now, however, this is still unknow (41). The opposite situation occurred within the highlands. The pandemic decelerated during the first months of the lockdown and that might be linked to a reduction on the speed of contagium among those cantons. For instance, and even though cumulative mortality in the highlands was critically high, the daily mortality was far below that seen on the Coast. Quito had less than 100 excessive deaths in a single day, while Guayaquil, the biggest coastal city in Ecuador, had more than 600 deaths a day (33).

Excess mortality is not only used for developing countries with poorer reporting systems. For example, in the United States, a 20% increase in deaths was reported during March–July 2020, and 28% during March–May (16), of which 67% corresponded to COVID-19-confirmed deaths (42).

In Italy, an increase in pandemic-related mortality was found, specifically related to an excess of deaths from undetermined respiratory illnesses (42, 43). Results consistent with the analysis by Ortiz-Prado et al. reported an increase in the number of deaths registered as acute respiratory distress syndrome during the first months of the pandemic in Ecuador while failing to provide accurate diagnosis (23).

In this sense, countries such as Italy or Spain, even though they were also struggling with an early violent COVID-19 first wave that took countries off-guard, had diagnosis capabilities that were superior than those reported in Latin America, including Ecuador (43). Michelozzi et al. reported that, in Italy, 52% of excess deaths were coded as COVID-19 (44). Another study in England showed that 23.6% of all deaths registered from February to June 2020 were registered as COVID-19 (30). Whereas in Ecuador, confirmed COVID-19 deaths only account for 3% of the total number of excess deaths during the first wave of the pandemic.

This difference between excess mortality and reported deaths from COVID-19 may be attributed to the country’s SARS-CoV-2 diagnostic testing strategy not being widely distributed, coupled with a congested healthcare system, especially during the highest volume of hospitalized patients (45).

The use of excess mortality can be the most reliable indicator to understand and estimate the real impact of the pandemic. This metric can also be used to imply how many people were infected during the early stage of the pandemic using reverse upscaling calculations as a proxy of the early attack rate within the country.

Finally, we believe that acknowledging the real impact of the pandemic using excess mortality will be useful to help inform public policy that will ensure future action toward prevention and health care service responses against future biological threats.

## 5 Limitations

The main goal of this study is to show how, using only the crude mean to compute excess deaths, the approach can be addressed erroneously. The reason why the estimates from the bootstrapped method are smaller than the classical method is because, when we use bootstrap, each simulation replicates the real distribution of data. In this way, it is possible to get an accurate value about the expected deaths and, as a result, a neat estimation of excess deaths. Using only the classical mean can lead to larger values, as seen in this paper. We think this is a strength of our method. The only limitation we can find is about computational time. The larger the series, the more time can be spent around simulations. We think both methods are comparable as they are statistical parameters, but they differ in the fact that classic mean only depends on some period of data whereas bootstrapped mean reaches stability due to the replication of distribution of deaths across simulations. The larger the number of simulations, the better quality of estimations.

The main limitation of this study is the use of one dimension to track excess deaths and bootstrapped excess deaths. A vast array of research has been conducted recently where excess deaths are also analyzed for other factors like age ranges, gender, and social strata (19). Unfortunately, the lack of data management in the official statistics unit of Ecuador has produced all death cases at an aggregated level, so there is not an official source to find more details about the impact of deaths across multiple strata. Another important limitation is the actual cause of deaths. There are countless deaths that were not certified as caused by COVID. Ecuador did not have Covid tests or methods to study the cause of death. Thousands of people were buried without any evidence. That was a widespread problem around the world, especially during the first few weeks of the pandemic.

Another limitation related to data quality is the level of death underreporting. After data analysis, it was quantified that there were delays between 3 and 5 days to register a death case at the official statistics unit. Using the common mean as the base for excess deaths tracker, considering this situation, can alter the results for excess because of extreme values that can appear on specific days. On the other side, using the bootstrapped mean helps to control the phenomenon of underreporting since this measure infers the data generation mechanism for the death cases series, thus better discriminating outliers. We also do not have information on age and sex, which is why we could not calculate the excess mortality for these two variables.

Additionally, having only the death case series for the country and provinces can impact the distribution of excess deaths below the traditional and bootstrap mean. Despite having data from 2017 to 2020 in terms of death cases, the absence of covariates like age or gender can influence the results profoundly. It could produce large, aggregated values, as our results show a difference of almost 9,000 cases between traditional excess and bootstrapped excess. This is related to how the distribution of excess deaths is affected because of necessary elements like socio-demographic variables and classification of death cases in reporting. This can be connected to the fact that another kind of information is needed, such as the results from similar illnesses like influenza and the contribution of effects from lockdown like the reduction of pollution (45). Further work is needed to determine the relative importance of these different factors on the overall estimates of excess deaths.

## 6 Conclusion

Ecuador had one of the highest numbers of excess deaths *per capita* in the world per day. The mortality excess rate shows that the SARS-CoV-2 virus spread rapidly in the country, especially in the coastal provinces of Santa Elena and Guayas during the first wave of the pandemic. These deaths reflect the number of active cases that were missing diagnosis but were responsible for the collapse of the health system during March and April 2020 in Ecuador. Due to the lack of diagnostic capabilities, excess mortality has demonstrated to be a good indicator of the real impact of the pandemic and can be used as a proxy to estimate the real attack rate that was greatly underreported.

## Data availability statement

The datasets presented in this study can be found in online repositories. The names of the repository/repositories and accession number(s) can be found at: https://github.com/covid19ec/DataDeathsEC.

## Author contributions

EO-P is responsible for the conceptualization of the study. RF-N contributed to data collection, data extraction, and data visualization. EO-P and RF-N were responsible for the elaboration of the first draft of the manuscript. EO-P, RF-N, JV-G, and JI-C contributed to the descriptive statistical analysis. EO-P, DC, JV-G, SL, and RL were responsible for the elaboration of the discussion section of the manuscript. JI-C and SL added important insights from a public health perspective. EO-P, RF-N, and JI-C were responsible for editing the final version of the manuscript. All authors contributed to the article and approved the submitted version.

## Footnotes

^1^https://www.ecuadorencifras.gob.ec/nacimientos-y-defunciones-informacion-historica/
